# Twelve tips for OSCE-style Tele-assessment

**DOI:** 10.15694/mep.2020.000168.1

**Published:** 2020-08-17

**Authors:** Raymond Lewandowski, Angela Stratton, Tarun Sen Gupta, Michelle Cooper

**Affiliations:** 1 The Australian College of Rural and Remote Medicine; 2 James Cook University and The Australian College of Rural and Remote Medicine

**Keywords:** tele-assessment, rural and remote medicine, high-stakes assessment, technology

## Abstract

This article was migrated. The article was marked as recommended.

The Australian College of Rural and Remote Medicine (ACRRM) has developed a flexible ‘tele-assessment’ approach to the delivery of its assessment modalities. Candidates can sit their examination remotely, close to their place of practice, which reduces the need for rural doctors - both candidates and examiners - to leave their communities for the purpose of assessment.

A major component of the assessment process is the Structured Assessment using Multiple Patient Scenarios (StAMPS) examination, which blends the formats of an Objective Structured Clinical Examination (OSCE) and a traditional viva vocè examination. It is a high-stakes assessment, that was designed to be academically rigorous, flexible, valid, reliable, and fair.

Since 2008 ACRRM has provided a videoconferencing option to candidates for their StAMPS examination allowing them to remain in or near their home location, while the examiners meet a central location. Travel restrictions due to the SARS-CoV-2 pandemic meant for the first time both candidates AND examiners participated in StAMPS via videoconference.

ACRRM conducted an online StAMPS assessment using videoconferencing technology for 65 candidates in mid-May 2020, with all candidates, examiners and support staff remaining in or near their home communities. These Twelve Tips outline some of the experience gained in providing tele-assessment over the past twelve years.

## Background

The Australian College of Rural and Remote Medicine (ACRRM) is the world’s first and only specialist medical college for rural medicine ( Smith *et al.*, 2007). A key principle in the design of ACRRM’s programmatic assessment has been flexibility in the delivery of the assessment modalities to allow ‘tele-assessment’ options for candidates to sit their examination remotely, close to their place of practice. This approach reduces the need for rural doctors - both candidates and examiners - to leave their communities for the purpose of assessment. The ACRRM Fellowship (FACRRM), the world’s only Fellowship examination in Rural Medicine, represents an accredited vocational endpoint that signifies certification as a specialist in rural and remote medicine ( Sen Gupta *et al*., 2020).

A major component of the assessment process, which is usually completed towards the end of training, is the Structured Assessment using Multiple Patient Scenarios (StAMPS) examination. StAMPS is a unique examination which blends the formats of an Objective Structured Clinical Examination (OSCE) and a traditional viva vocè examination. StAMPS scenarios reflect real life situations and provide an opportunity for the candidate to explain the rationale behind their thinking ( Wilkinson *et al.*, 2008). It is a high-stakes assessment, that was designed to be academically rigorous, flexible, valid, reliable, and fair ( Australian College of Rural and Remote Medicine, 2020).

Since 2008 ACRRM has provided a videoconferencing option to candidates for their StAMPS examination in addition to face-to-face. In both formats candidates stay in one place (on a videoconference link or in one room) and the examiners rotate between rooms. However, due to the SARS-CoV-2 pandemic, neither candidates nor examiners were able to convene in an examination centre, therefore necessitating for the first time the need for both candidates AND examiners to participate in StAMPS via videoconference.

As a result, ACRRM conducted an online StAMPS assessment using videoconferencing technology for 65 candidates in mid-May 2020. All candidates, examiners and support staff remained in or near their home communities obviating the need for travel and minimising the risk to candidates, examiners and staff, and their communities.

ACRRM’s StAMPS examination consists of eight ten-minute stations, each with a number of sub-components and each delivered by a different examiner. As with other OSCE-style examinations there is a timetabled rotation around each of the stations so that at the end of the examination each candidate has seen each station and each examiner. There may be more than one such circuit running in parallel, and circuits run sequentially to accommodate all of the candidates.

The May 2020 StAMPS examination utilised two cycles of eight examiners each, supported by a number of administrative and technical staff. There were some modifications to the process and the timing as detailed below, but the content remained largely unchanged compared with the standard examination. ACRRM’s other assessment modalities can also be undertaken at a distance ( Sen Gupta *et al*., 2020), meaning that it has continued to offer uninterrupted assessment and training throughout the pandemic, uniquely among Australian Medical Colleges.

Over the past twelve years ACRRM has developed extensive experience in tele-assessment both prior to social-distancing and with subsequent adaptations for the COVID environment. This paper describes Twelve Tips for delivering high-stakes OSCE-style examinations via video tele-assessment, using the recent example of the Structured Assessments using Multiple Patient Scenarios (StAMPS) examination.

## Context

Donald Berwick has described the severe acute respiratory syndrome coronavirus 2 (SARS-CoV-2) as a stern teacher, noting, ‘Answers to the questions it has raised may reshape both healthcare and society as a whole’ ( Berwick, 2020).

These are indeed unprecedented times, with many challenges to healthcare worldwide, as well as to medical education. Rose observes that, ‘The need to prepare future physicians has never been as focused as it is now in the context of a global emergency’ ( Rose, 2020). Considerable attention is, quite rightly, being directed to the challenges of maintaining safety - for the patient, the student, and the system. However, clinical teaching along with assessment of students at all levels must also be considered in the face of a system under considerable strain. Indeed, given the imperative to produce a workforce in a setting where clinical experience may be limited, it may be even more important than usual to get the assessment right in order to assure patient safety and public confidence in standards.

ACRRM has considerable experience in ‘tele-assessment’, having designed its assessment processes to be delivered to rural and remote candidates in or near their home location. These experiences, as outlined in the following Twelve Tips, may therefore be useful to educators in other jurisdictions, who are looking to conduct assessments in settings where social distancing requirements and travel restrictions may make ‘traditional’ assessments infeasible. The tips are relevant to an OSCE-style assessment where candidates (or examiners) rotate in a circuit, which may be one of a number run in parallel.

## Tip 1: Assess what is assessable

Revise your assessment blueprint and tailor the examination to the tele-assessment format. Multiple physician competencies can be covered in oral examinations conducted by videoconference - history, communication skills, diagnosis, clinical interpretation, management, explanation, and ethical/professional issues can all be explored - and assessed ( Jefferies *et al*., 2007). Think about what can be best tested or demonstrated in your chosen format without limiting yourself by what has ‘always been done’.Possibilities are limitless, ingenuity rules.

Blueprint the stations in order to map which curricular components can be best assessed in the oral format, then think about how to assess gaps that may be missing from the change in assessment structure. ‘Knows’ level questions are likely better suited to written assessment, therefore reserving the higher ‘knows how’ or ‘shows how’ questions for tele-assessment. Physical examination is going to be a challenge to assess by tele-assessment, however, could be assessed through other modalities like work-based assessment. Examples include the mini-CEX, or direct observation by use of distance means, with the examinee wearing a Go-Pro™ or similar, with appropriate attention to patient consent and privacy.

## Tip 2: Design the examination to match the format

Carefully consider your examination construct. As with all OSCE-format exams, formally plan the rotations. The rotation plan will need to take into account the details of your Information Technology (IT) platform and how participants will interact. Developing the plan requires undertaking the calculations to factor in the number of scenarios, reading time and briefings. The number of scenarios in the examination provides a good guide as to the size of each virtual ‘group’, although it is possible to include rest stations. The recent ACRRM StAMPS examination consisted of eight scenarios with one examiner per scenario. We therefore formed two ‘virtual groups’ of eight examiners each with an additional examiner as team Lead and Quality Assurance (QA). During each rotation, eight or nine candidates were examined, using a rest station as required. We ran two rotations per day for each virtual group.

**Figure 1.**
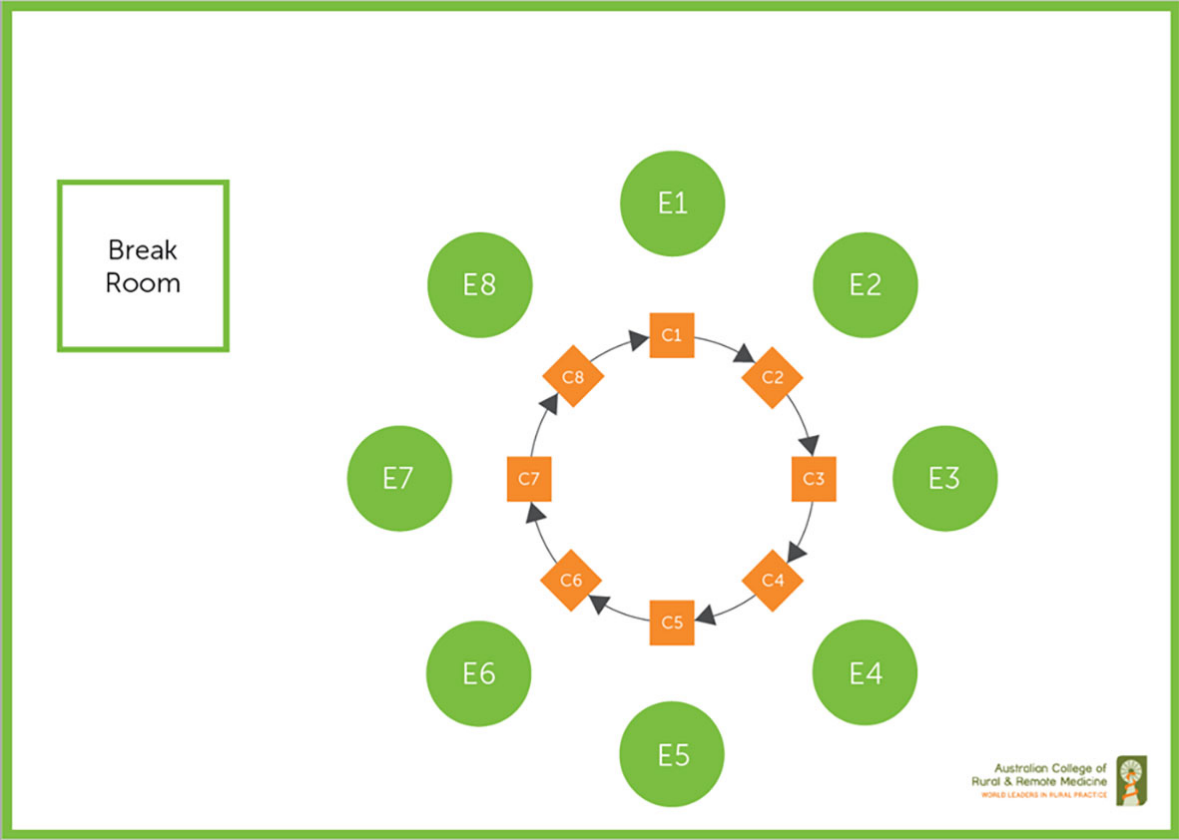
Traditional OSCE-style examination.

**Figure 2.**
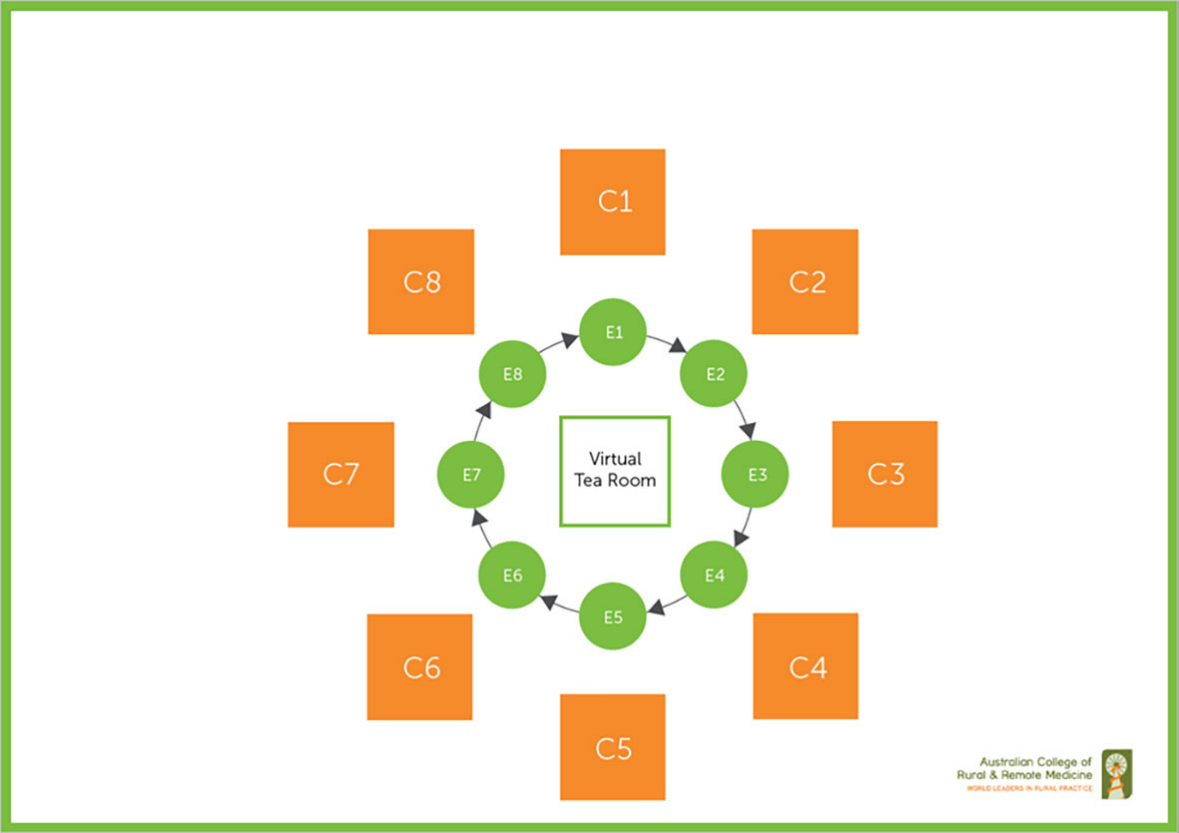
Virtual StAMPS examination.

Figure 1 illustrates the traditional OSCE-style examination. Examiners stay in one place for the duration of the examination while candidates move through each station in turn. The physical break room or meeting point for examiners is external to the rotation plan. Figure 2 illustrates the virtual StAMPS examination. Candidates stay in their individual virtual room while Examiners move through each virtual room in turn. Examiners can enter the virtual tea-room between cases to discuss issues and for quality assurance.

Examiners (E) stay in one place for the duration of the examination while candidates (C) move through each station in turn.

Candidates (C) stay in their individual virtual room while Examiners (E) move through each virtual room in turn.

Think carefully about the timing of the stations. In the case of the recent examination we allowed the standard timing for each station, however the time *between* stations was increased from the usual 5 minutes to 10 minutes. This change enabled additional time for inter-examiner discussions in the ‘virtual tea-room’, plus the logistics of completing the paperwork and submitting it online. The increased time between stations also allowed for a short buffer for IT delays while the examiner entered the virtual room. Any other delays could be made up at the end of the cycle to ensure the candidate received the full allotment of time.

## Tip 3. Prepare the cases

Prepare clinically relevant written scenarios that enable the candidate to have all relevant materials available during reading time. ACRRM’s StAMPS examination avoided stations that require handouts as logistically this is challenging and can lead to invigilators giving the wrong sheet and/or at the wrong time, or delays while the correct handout is found. An alternative may be to provide information via a ‘screen share’ style function, factoring in examiner IT skillset and additional time or bandwidth capability.

Be mindful of the length of oral questions. It may be tempting in the absence of handouts to try to provide significant clinical information in an evolving or sequential style case, however there is a risk of sound quality concerns that make numbers or fine details easily mis-heard. The same principle applies for the complexity of language used in the questions. All StAMPS scenarios were ‘road-tested’ virtually with a recent successful candidate and edited as required.

Authentic scenarios can be written with simulated patients - or colleagues (eg handing over care of a palliative care patient; discussing assessment and management of a sick child with a remote area nurse; arranging retrieval of a critical patient with an aeromedical service). The recent rise of tele-consultations has created a demand for clinical reasoning skills in the absence of physical examination, and this is ideally suited to being assessed in tele-assessment.

## Tip 4. Prepare the examiners

Ensure examiners are well briefed and familiar with the process and the IT platform used. ACRRM has long used ‘moderator sessions’ as a form of ‘tele-examiner training’. Pairs of examiners meet ahead of time to discuss and practise their cases with a moderator present. All examiner practice and training for the recent StAMPS examination was conducted on the IT platform used for the actual examination.

Before the examination, run a virtual examiner briefing as a large group, with smaller on-the-day catch-ups as needed. An examiner debrief at the end of the day is also helpful. Consider having a virtual ‘tea-room’ and/or central point of contact where examiners can catch up with each other and the staff between cases. Remind examiners about minimising the risk of potential interruptions during the examination plus consideration of an ‘appropriate virtual background’ for their video. Ensure phones are set to ‘do not disturb’, notifications on the computer being used for the examination are turned off, and people and pets are not going to be moving in and out of the room being used.

It may also be worth emphasizing the benefits of this virtual examination when recruiting examiners. Our experience is there is reduced examiner fatigue due to no travel requirements. Most ACRRM examiners live and work rurally and would have to travel to a test centre for a face-to-face examination. There is also less time away from work, so examiners are still able to attend to clinical commitments like ward rounds and to family responsibilities.

## Tip 5: Prepare the Candidates

Clear communication with candidates is vital. They need to understand the plan and what it means for them and their preparation. In the case of the recent StAMPS examination run during the SARS-CoV-2 pandemic, candidates were given the opportunity to postpone without penalty. While a number chose to do so, many were grateful for the chance to be able to progress their training without interruption.

A clear communication plan, and the ability to opt in or out is an important strategy in ensuring acceptability for candidates and getting them to ‘buy in’. A practice session using the IT platform and the actual computer they will use on the day is essential to troubleshoot and ensure familiarity. ACRRM conducted multiple open sessions on the platform for candidates in the weeks ahead of the examination to allow testing, familiarization and to ask questions.

Like the examiners’ feedback, feedback from ACRRM’s recent candidates has been favourable. They reported reduced fatigue from travel and found the examination a more relaxed experience due to sitting in a familiar environment rather than face-to-face with the examiner away from their home location.

## Tip 6: Get the IT Platform right

The choice of platform will depend on several factors including scale of the examination, cost, technical requirements, reliability, security features and IT support. Above all it needs to be easy to use for examiners and candidates. Consider the number of accounts you require - separate accounts means not everything shuts down if one account fails.

Consider how many ‘rooms’ you will need and if your candidates will stay in one ‘virtual room’ or whether ‘break-out’ rooms are used for a larger group waiting room. In the recent StAMPS examination candidates stayed in the same room, with examiners entering and leaving at appropriate times managed by the room monitor. Skilled IT support on the day is vital to quickly manage any difficulties, allowing examiners and candidates to focus fully on the actual examination content.

The Zoom™ platform was used for several reasons: it was web-based (therefore no software to download) and used substantially less bandwidth, which is important in rural and remote locations. The platform permitted sufficient administrator rights to other levels of users so that on the day of the examination support staff could rebuild or correct challenges without the need for the IT services team, and without affecting other users on the system. This meant that if a particular virtual room had an issue the environment could generally be rebuilt by the local team rapidly, and without having to reassign access to a different user. Zoom™ also allowed a number of features to be customized to maximize security, as set out in Tip 7.

## Tip 7: Be obsessive about security

Careful attention to security is needed at every step of the process. Candidates in remote sites will need approved invigilators who are able to confirm their identity and ensure there are no breaches of examination process such as accessing unauthorised material and examination confidentiality is maintained. Examiners require a secure process for receiving, storing, and submitting marking sheets. For the recent StAMPS examination, traditional printable material was used by both candidates and examiners. Invigilators were responsible for candidate materials, while examiner ‘packs’ were collated and sent via registered mail. It was thought that online documents would create added complexity in the new environment, but this is being explored for future exams.

Use passwords to enable access to the examination and waiting rooms to ensure appropriate access. Central support staff, or room monitors, should scrutinize all rooms to ensure only authorised personnel are present. Check that the software does not allow candidates to change their identity. Consider recording the entire session from the initial introduction of the invigilator to the destruction of the examination materials at the conclusion.

Zoom’s™ security features also matched ACRRM’s specifications. Data was routed through Australian servers and examination content was prepared, managed, and stored outside of Zoom™ on ACRRM’s usual servers, i.e. Zoom™ was only used to deliver the examination experience. Screen sharing and desktop functionality was turned off for candidates by default to prevent the opening of additional windows to access information or share content. Virtual backgrounds and private chats were also disabled to ensure additional privacy. Real-time room monitoring by trained staff, unique meeting IDs, password protection and the use of virtual waiting rooms were all used to prevent unauthorised access.

## Tip 8: Information Technology: Test, test, and test again

Test the testable things, then test them again! Candidates and examiners will need instruction on the requirements for the examination such as appropriate reliable bandwidth, video capabilities, microphone, and speakers. In our experience a headset is preferable to reduce sound feedback, but this needs to be tested as well. Just like testing the IT platform, all participants need opportunities to check and troubleshoot their systems prior to the day. Prior to StAMPS ACRRM ran a series of separate training sessions for both the examiners and candidates, enabling questions to be resolved and staff to identify candidates who may need extra support on the day. A future consideration will be to run the ‘Mock exam’ in the same virtual format to provide a more authentic training experience and identify any IT issues well ahead of time.

## Tip 9: Develop a Quality Assurance (QA) process

Quality assurance processes should address delivery and marking of the examination. For the StAMPS examination multiple levels of QA were employed. There were two Lead examiners (Lead) who participated in the moderation sessions for all of the stations, one Lead for each group of examiners. The Leads observed the station delivery in each room during every rotation, observing each scenario on average once per rotation. The Leads then met with the actual examiners from their group in the ‘tea-room’ directly after each observed station to discuss delivery consistency and marking.

The Zoom™ platform allowed for the recording of the entire examination. After completion of the exam, the Leads reviewed videos of examiners that were not in their group looking for consistency in delivery and ensuring that marks were appropriately attributed for answers given. As the entire examination was recorded for each candidate as a single file (approximately 160 minutes), there was some time lost in identifying specific stations in the examination. Future developments will include a system for developing time-markers to locate individual stations more rapidly in each video.

## Tip 10: Develop your marking process

StAMPS has a standard marking system for the examination. This system was employed for the virtual examination and was reviewed with all examiners in advance at the moderation sessions. The paper examination materials used by the examiners to deliver the examination included a separate marking sheet and set of marking items for each candidate, as well as a rotation plan and standard examination materials such as incident reporting sheets. All materials were returned to ACRRM by prepaid registered post.

To keep the outcome data and review process timely it was important for the administration team to receive the marks for the candidates on the day of the examination. To accomplish this, examiners were asked to complete their marking sheets, photograph them, and email them to the examination team. The team then followed up with each examiner at the end of the rotation regarding any marking sheet that they had not received. As noted in Tip 7 above, electronic marking systems would streamline this collating process and is being considered for the future.

## Tip 11: Lights! Camera! Action! Get the station logistics right

The StAMPS examination is designed with the expectation that each candidate will have ten minutes to complete the questions for each scenario. It is critical that this time be allotted to them so efforts must be made to preserve it. The platform used for the virtual examination requested the examiner to choose both the audio and video source to be used every time a room was entered, the examiners were also expected to keep track of time which required starting a timer. These tasks took time to perform but were not intended to take assessment time from the candidate.

For the recent StAMPS examination, a PDF file was sent to all examiners with sequential links and times for each of the candidates they would be examining.

The most streamlined and foolproof method seemed to be for the examiner to click on the link for the candidate a minimum of thirty seconds early. The examiner would enter a waiting room and the room monitor would then allow them into the examination room approximately ten seconds before the start time. This allowed the examiner time to get all the technical necessities sorted out prior to the start of the scenario. Being let into the examination room too early was not advantageous as the examiner appeared on the examinees’ screen which could be awkward and distracting. The examiner was expected to confirm the candidate’s name and the anticipated scenario number as a final check before delivering the scenario. The room monitor was also empowered to ensure that ten minutes were allotted for the scenario. Should the session start late it would also be allowed to finish late.

If additional people were present in the room for the purpose of QA or pre-approved observation, they were instructed to keep both cameras and microphones off.

Backgrounds were disabled for candidates as an additional security measure. Examiners were allowed to use backgrounds providing they had been used during their test of the system to ensure that it did not interfere with their upload speed and were not distracting to candidates.

## Tip 12: Plan to fail

There is a common adage ‘failing to plan is planning to fail’. The contrapositive is not true, however. If you plan to fail, then you are prepared for the failure and ready in advance to work around it. Develop a formal risk management document - and share it with decision makers and other stakeholders.

The biggest risk in the recent StAMPS examination was on the technical side. A number of plans were in place for technical failures. Mobile phone numbers for all examiners and examinees were distributed to examination leads. Emergency numbers were also distributed to all examiners and examinees in case of platform-related failures. It is critical that there are enough people at the host site to answer the ‘phones. There was an anticipation that technical issues would be mostly ‘self-correcting’, but this still takes time away from examinees. These issues would include link failure, late entry into a room and similar problems. Room monitors were instructed to allow extra time as required before ending a scenario if this occurred. There was also time at the end of each rotation where up to two make-up scenarios could be given to each candidate if major issues occurred during the normal time. IT staff were available in real time to assist with troubleshooting both ends of the connection. Other back-up systems included the use of a telephone (teleconference system) and reinstatement of the chat function in case of a loss of audio.

There were also non-IT failures that were anticipated. The examination materials were paper based and so delivery to the examiners and examinees was dependent on the postal service. An online portal with all necessary materials was also made available to examiners and they were asked to ensure they could print these materials if their examination packs did not arrive by the day prior to the exam. The fact that examiners administered the examination from remote locations presented another potential failure in that if an examiner was not able to turn up it would not be recognised until the examination began. A pre-examination meeting was therefore held each day, in which each examiner could be accounted for and all technical issues could be identified and solved prior to the examination beginning.

Given the high-stakes nature of this assessment, and relatively short planning time in a rapidly changing environment, the ACRRM team adopted a KISS - Keep It Simple - strategy. A number of enhanced features could have been utilized such as screen sharing of questions, virtual handouts, online marking, and timers. However, these also carried more risk, so the decision was made to opt for simpler user-friendly options for this examination. With increasing experience and confidence there are many more refinements that can be introduced.

## Conclusion

With current readily available technologies many assessments that traditionally were performed in person can now be performed via tele-assessment improving convenience, reducing travel time and costs, and allowing adaptation to necessities and impediments outside of the examination itself. ACRRM has shown that a high-stakes OSCE-style assessment can be successfully offered via a virtual format. Given travel restrictions in the recent pandemic it would have been near-impossible to mount a face-to-face examination, so this approach enabled uninterrupted continuation of training and assessment.

Post hoc evaluation indicates that this approach was acceptable to candidates, examiners and other stakeholders, and examinee performance was comparable to face-to-face offerings for this assessment.

As is true for all assessment modalities, we suggest the ‘3 Ps of assessment’ - preparation, preparation, and preparation - are vital for success. These Twelve Tips should help direct this preparation for other educators who may need to adapt their assessments for virtual delivery. While these approaches were developed for Australia’s rural and remote context the lessons learned may be applicable in any setting. This may well be the new normal.

## Take Home Messages

• High-stakes OSCE-style tele-assessment is possible using current technology, even in rural and remote settings
• Many curricular components can be assessed in this way
• Careful preparation, attention to detail and vigilance with security is vital
• This approach is acceptable to candidates, examiners, and other stakeholders

## Notes On Contributors

Raymond Lewandowski is StAMPS Lead Examiner, The Australian College of Rural and Remote Medicine. He is a Senior Medical Officer at Innisfail Hospital, Queensland and is the President of the Rural Doctors Association of Queensland.

Angela Stratton is StAMPS Lead Reviewer, The Australian College of Rural and Remote Medicine. She is a Rural Generalist at Mt Beauty Medical Centre, and Medical Educator with Murray City Country Coast General Practice Training.

Tarun Sen Gupta is Chair of the Assessment Committee, The Australian College of Rural and Remote Medicine, and Professor of Health Professional Education at the James Cook University College of medicine and Dentistry. ORCID: https://orcid.org/0000-0001-7698-1413

Michelle Cooper is General Manager, Member Services, The Australian College of Rural and Remote Medicine.
