# Supplementary material for: Twelve tips for OSCE-style Tele-assessment
Source: MedEdPublish (2016). 2020 Aug 17;9:168. [Version 1] doi: 10.15694/mep.2020.000168.1 (PMC10702649; doi:10.15694/mep.2020.000168.1)
Supplement: Peer review history

# Reviewer report: Reviewer response for version 1

**DOI:** 10.21956/mep.20134.r31085

**Published:** 2020-08-31

**Authors:** Ronald M Harden

**Affiliations:** 1 AMEE

This review has been migrated. The reviewer awarded 4 stars out of 5 The ACRRM are to be congratulated on implementing a practical approach to clinical assessment at a distance at this time with a COVID pandemic. The 12 Tips provided highlight the experience gained and the thought put into it. There are clear lessons to be learned which are relevant in different OSCE scenarios and this is a recommended read.I would have welcomed the authors thoughts on the feedback to candidates, on pass fail decisions and remediation , to how patients are best represented eg a slide, a video clip or a simulator and how the results and WBA is intergrated

Reviewer Expertise:

NA

No decision status is available

# Reviewer report: Reviewer response for version 1

**DOI:** 10.21956/mep.20134.r31084

**Published:** 2020-08-25

**Authors:** Tim Wilkinson

**Affiliations:** 1 University of Otago,Christchurch

This review has been migrated. The reviewer awarded 5 stars out of 5 A nice paper drawing on extensive hands-on experience over many years in running ‘tele-assessment’. It is now very topical, so drawing on such experience should help others.I was initially confused by tip 1 – ‘assess what is assessable’ as that is a trap that many fall into inadvertently – ‘we’ve got a MCQ test, now let’s work out what we can assess’. As opposed to assess what you value. However, what the authors mean is only try to assess those things that are suited to that format - so maybe it is better reframed as ‘assess what is assessable using this format’. Or more proverbially – ‘horses for courses. The consequence of this is then to suggest don’t try to assess things that won’t use that format. Which then also leads on to perhaps another tip - once you’ve worked out what can’t be assessed then think about how you might fill those gaps with different assessment tools. Maintaining the overall assessment programme blueprint should always be the goal.

Reviewer Expertise:

NA

No decision status is available
